# Extracting Medical Information From Unstructured Clinical Text Using Large Language Models to Enhance Health Care Interoperability: Proof-of-Concept Study

**DOI:** 10.2196/92413

**Published:** 2026-07-02

**Authors:** Bahadır Eryılmaz, Kamyar Arzideh, Mikel Bahn, Hendrik Damm, Sina Warmer, Henning Schäfer, Ahmad Idrissi-Yaghir, Tabea M G Pakull, Lea Jessica Albrecht, Jens Kleesiek, Georg Lodde, Christoph M Friedrich, Elisabeth Livingstone, Dirk Schadendorf, Katarzyna Borys, Felix Nensa, René Hosch

**Affiliations:** 1 University Hospital Essen, Institute for Artificial Intelligence in Medicine (IKIM), Girardetstraße 2, Essen, NRW, 45131, Germany; 2 University Hospital Essen, Institute of Diagnostic and Interventional Radiology and Neuroradiology, Essen, NRW, Germany; 3 Central IT Department, Essen, NRW, Germany; 4 Department of Computer Science, University of Applied Sciences and Arts Dortmund, Dortmund, NRW, Germany; 5 University Hospital Essen, Institute of Medical Informatics, Biometry and Epidemiology, Essen, NRW, Germany; 6 University Hospital Essen, Institute for Transfusion Medicine, Essen, NRW, Germany; 7 Department of Dermatology, University Hospital Essen, Essen, Germany

**Keywords:** large language models, artificial intelligence, AI in health care, interoperability, Fast Healthcare Interoperability Resources, FHIR, entity extraction, generative AI

## Abstract

**Background:**

Unstructured clinical text remains a major barrier to interoperable data reuse and large-scale secondary analysis in health care. Large language models (LLMs) have the potential to automate the extraction of structured clinical information; however, their application is limited by the scarcity of high-quality annotated training data.

**Objective:**

To address these limitations, this study aims to develop and validate a scalable, privacy-preserving framework that uses synthetic data generated from structured Fast Healthcare Interoperability Resources (FHIR) to fine-tune open-source LLMs for the effective extraction of interoperable clinical information from unstructured text.

**Methods:**

We evaluated an LLM-based framework for extracting structured clinical information from cancer-related discharge letters and mapping it to representations compatible with FHIR. To enable large-scale supervised training, we developed a random sample generator that creates synthetic discharge letters using Qwen3-235B by randomly sampling and aggregating structured FHIR data from 41,175 patients with cancer. The resulting synthetic discharge letters (n=75,000) were paired with their originating structured data, forming a large-scale dataset for fine-tuning MedGemma 27B, a 27-billion-parameter medical language model. Evaluation was conducted on the synthetic test dataset (n=7500), real-world discharge letters (n=30), which were evaluated by physicians and a medical student, and a comparative one-shot approach using open-source models (Qwen3, LLaMA, and GPT-OSS).

**Results:**

The fine-tuned model achieved high extraction performance across multiple clinical entities on the synthetic test set, with *F*_1_-scores of 0.84 for full *International Classification of Diseases* diagnosis codes, 0.99 for tumor-related information, 0.99 for laboratory values, 0.99 for medication names and dosages, and 0.94 for Anatomical Therapeutic Chemical medication codes. The extraction of procedure-related information was more challenging, with *F*_1_-scores of 0.63 for OPS codes and 0.90 for procedure descriptions. The fine-tuned model consistently outperformed general-purpose LLMs in a one-shot comparison across nearly all extraction categories. When evaluated by physicians on real-world discharge letters, the model achieved case-level correctness rates of 78.9% for *International Classification of Diseases* diagnoses, 86.1% for tumor-related information, 93.0% for medications, and 61.3% for procedures.

**Conclusions:**

These results demonstrate that synthetic text generation from structured clinical data enables the effective and scalable training of LLMs for extracting interoperable, multientity clinical information from unstructured documentation.

## Introduction

Electronic health records (EHRs) are popular in modern health care, serving as digital repositories of patient events, diagnoses, procedures, and observations. They are relevant not only for clinical care but also for health care operations, quality management, and medical research [1]. However, processing EHR data remains a complex challenge [2], particularly the unstructured portion, which constitutes approximately 80% of all EHR data [3]. Clinical narratives, such as discharge letters and progress notes, are rich in contextual detail but are difficult to reuse due to a lack of standardization, high linguistic diversity, and strict privacy considerations [4]. These issues limit the ability to extract and leverage valuable information from free-text clinical documentation efficiently. In Germany, hospitals generate vast volumes of unstructured EHR data each year [5]. For example, based on a query of the hospital information system conducted in 2025, the University Hospital Essen produces approximately 140,000 discharge letters and 2.9 million progress notes annually. Unstructured clinical text often contains richer and more nuanced information than structured data, underscoring its potential value for secondary use [6]. Consequently, transforming unstructured data into structured, machine-readable formats has become a primary focus of medical informatics [7].

To achieve this structural transformation effectively, the domain has increasingly converged on the Health Level Seven Fast Healthcare Interoperability Resources (FHIR) standard [8]. Unlike legacy formats, FHIR uses a modern, web-based approach to represent clinical data as granular, independent *resources*, such as *Conditions*, *Procedures*, or *Observations*, enabling seamless data exchange and standardized representation. Standards such as FHIR are pivotal for achieving semantic interoperability, ensuring that medical information extracted from isolated notes is universally understood by downstream applications and research platforms. This development is further reinforced by large-scale initiatives such as the German Medizininformatik-Initiative [9] and the emerging European Health Data Space [10], both of which position FHIR as a central interoperability standard for cross-institutional data sharing and secondary use of health data. However, while FHIR provides the necessary framework for interoperable health care data, automatically extracting relevant information from clinical documents remains an open issue. Early work by Li et al [11] introduced FHIR-GPT, a framework leveraging large language models (LLMs) to convert unstructured clinical narratives into standardized FHIR resources, demonstrating that LLM-based approaches can enhance health data interoperability without relying on complex, multistep natural language processing (NLP) pipelines.

At the same time, recent advancements in NLP, particularly the rise of LLMs, have introduced promising new capabilities for processing unstructured medical text [12-18]. A generative approach by Majid et al [19] compared encoder-only architectures against generative LLMs in a cohort of ophthalmology patients, demonstrating that modern generative models can achieve superior performance in extracting named entities from unstructured medical reports. Recent initiatives [20,21] have focused on integrating open-source LLMs into German clinical workflows to enhance information extraction. Despite these promising advancements, the field continues to grapple with significant challenges: the scarcity of training data and the prohibitive time and effort required for the manual evaluation of unstructured clinical text. To address these bottlenecks, synthetic data generation has emerged as a crucial strategy, increasingly recognized as a powerful solution to overcome data scarcity in clinical NLP [22-24].

In this work, we introduce PIGEON (Patient Information Generation from Organized Notes). Rather than presenting merely another fine-tuned extraction model, PIGEON establishes a scalable paradigm for generating synthetic supervised training data directly from interoperable clinical backends (FHIR). This approach enables the robust fine-tuning of open-source LLMs under strict data governance constraints. We demonstrate this framework by fine-tuning MedGemma 27B using data from a production FHIR R4 server containing over 2 billion resources. We have validated PIGEON’s efficacy through synthetic dataset benchmarking and assessed its real-world performance via clinician review of predictions on real-world discharge letters.

## Methods

### Ethical Considerations

This study was approved by the Ethics Committee of the University Hospital Essen (approval number 24‐12111-BO). Due to the study’s retrospective nature, the requirement for written informed consent was waived by the Ethics Committee.

### Discharge Letter Generator

**Figure 1.**
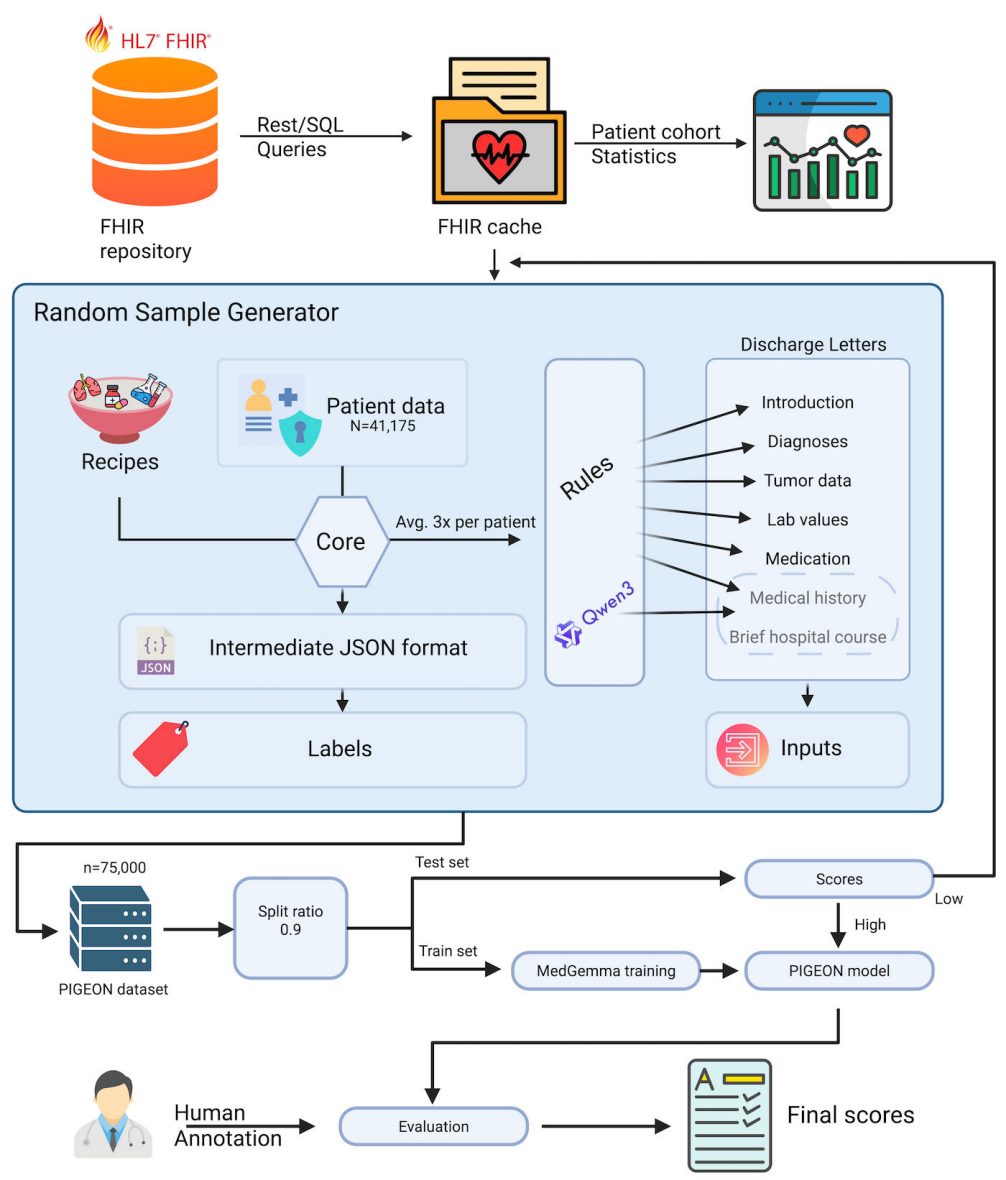
Schematic overview of the PIGEON (Patient Information Generation from Organized Notes) workflow for extracting clinical data. The process begins with querying patient cohort statistics from the Fast Healthcare Interoperability Resources (FHIR) repository to populate a local FHIR cache. The “Random Sample Generator” then creates a synthetic training dataset by pairing structured patient data (transformed into an intermediate JSON format/Labels) with synthetic clinical narratives (“discharge letters”) generated via Qwen3 and recipes. The resulting PIGEON dataset is split (0.9 ratio) to fine-tune the MedGemma 27B model. The framework includes a validation feedback loop to refine generation based on test scores, concluding with a final performance evaluation against human-annotated real-world data. SQL: Structured Query Language.

To generate input-output pairs from the FHIR cache, we used a synthetic discharge letter-generation process with the Qwen3-235B LLM [25]. This approach used the FHIR cache to create realistic discharge letters by selecting relevant FHIR resources and formatting them into coherent document sections. The goal was to simulate the unstructured nature found in authentic discharge letters while maintaining control over the content and structure. The complete workflow architecture is illustrated in Figure 1.

**Figure 2.**
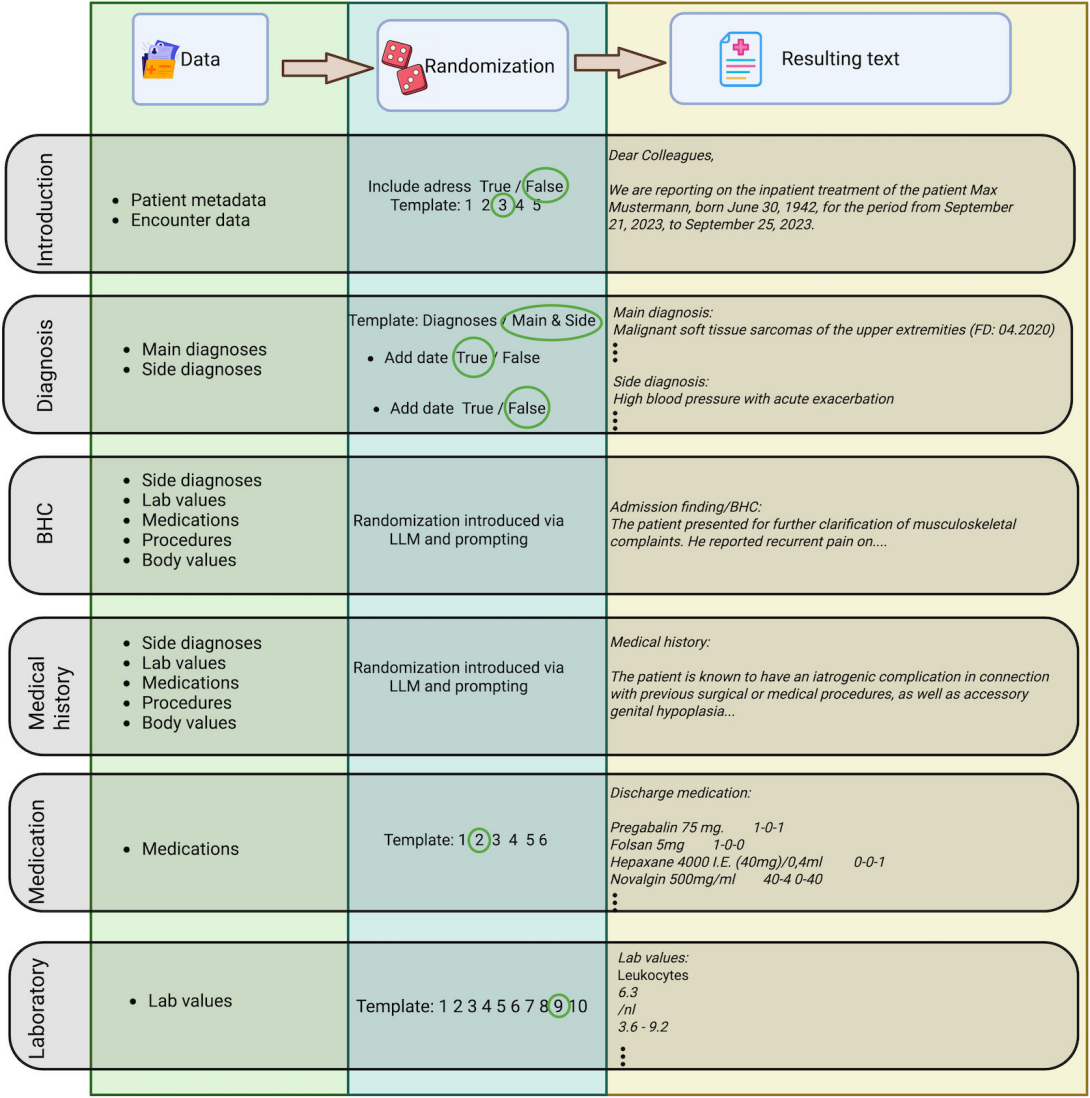
Detailed schema of the synthetic text generation pipeline. The workflow transforms structured input data (left column) into coherent synthetic discharge letters (right column) through a randomized logic layer (middle column). For structured sections such as Introduction, Diagnosis, Medication, and Laboratory, the system uses the “Random Sample Generator” to select from predefined templates and toggle specific parameters, such as the inclusion of dates or addresses. Conversely, narrative-heavy sections like the Brief Hospital Course (BHC) and Medical History are generated via large language model (LLM) prompting to introduce linguistic variability and simulate realistic free-text reporting.

Following this, a synthetic dataset, hereafter referred to as the PIGEON dataset, is generated from this cache using the random sample generator. The described workflow followed an iterative approach, allowing the refinement of the data selection rules and optimization of the instructional prompts. This generator uses a scalable, class-based random selection of FHIR resources to produce synthetic discharge letters that closely resemble authentic clinical correspondence. This random selection happens through rule-based templates (recipes), which tell the generator exactly which data to use for generation. Crucially, to preclude any risk of memorization or structural leakage from real patient records, no actual discharge letters were used as prompts within this framework. Instead, all narrative variability is strictly derived either through rule-based recipes or via LLM generation conditioned exclusively on the structured FHIR data. In total, 149,000 recipes were derived from the available FHIR cache. A detailed illustration of this process and the randomization steps is demonstrated in Figure 2.

To ensure data consistency across input-output pairs, multiple recipes are generated per patient. The generator accepts a recipe as input, retrieves the corresponding data, and constructs specific sections using a set of structural and content rules and the Qwen3-235B vLLM end point. Ultimately, the generator produces both the associated labels and the final synthetic discharge letter.

### Synthetic Discharge Letter Structure

The synthetic letters were generated by reading the recipe information and the selected FHIR resources from the FHIR cache and mapping them to predefined sections. Each section was populated using relevant resource types, as outlined in Table 1.

**Table 1.** Semantic mapping of structured FHIR^a^ resources to defined sections of the synthetic discharge letters.^b^

Letter section	Relevant FHIR resource types	Type of medical data
Greeting	Patient, Encounter	Patient information and stay duration
Main Diagnosis	Condition	Diagnose and date
Side Diagnosis	Condition	Diagnosis and date
Tumor Data	Condition, Procedure, Observation	Comprehensive tumor documentation
Anamneses	Condition, Procedure, Observation, Medications	Diagnosis, procedures, lab values, vital signs and body information, medications
Clinical Course	Condition, Procedure, Observation, Medications	Diagnosis, procedures, lab values, medications
Lab	Observation	Lab values
Medications	Medication, MedicationStatement	Medications, medication dosages

a FHIR: Fast Healthcare Interoperability Resources.

b The table shows the specific resource types used to populate each narrative component, ensuring that clinical data elements (eg, diagnoses, medications, procedures) are accurately represented in the generated documentation context.

**Figure 3.**
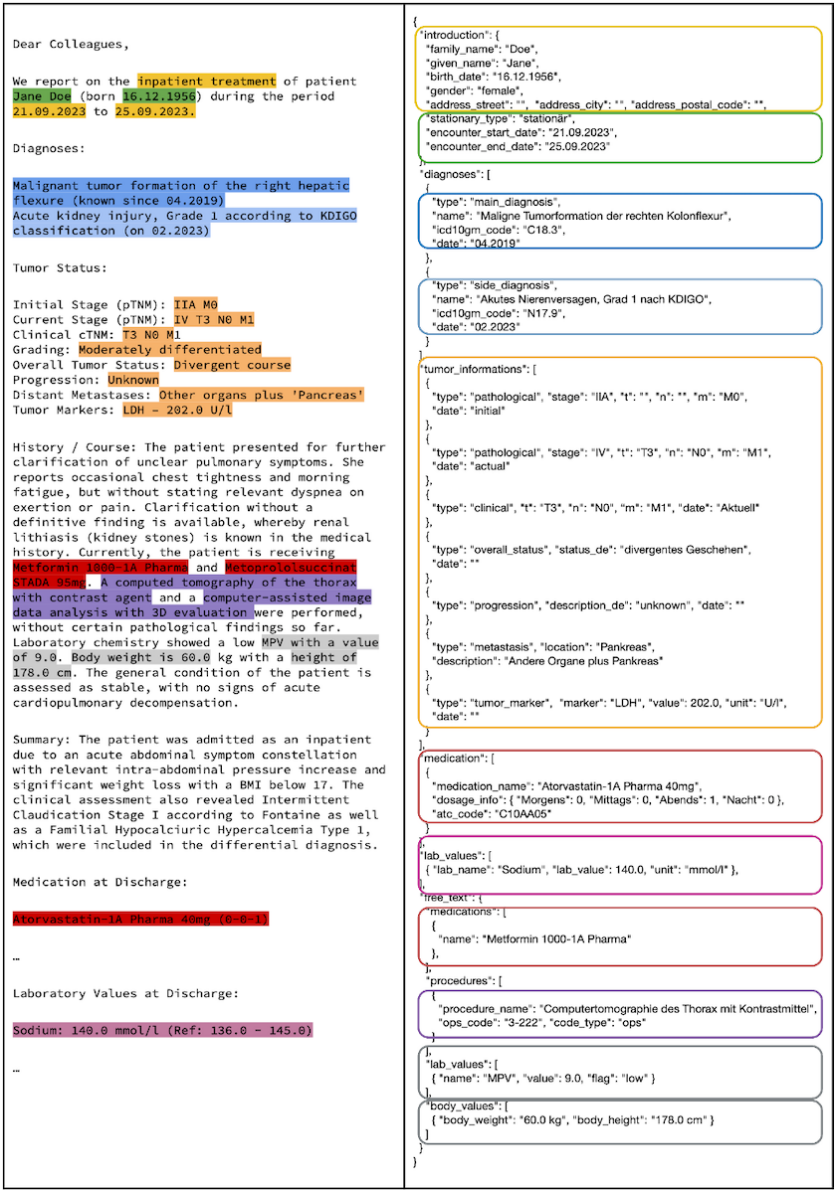
Anonymized and translated discharge letter with output from the model side-by-side. Extracted entities are color-coded. For demonstration purposes, the letter and JSON are cut, and other entities extracted by the model are not shown. These excluded entities include medications, lab values, and diagnoses.

To introduce variability and enhance sample diversity, multiple randomization strategies were applied during resource selection and letter composition including generating multiple variants of diagnoses, several templates for non-LLM sections, previously explained recipe logic, and dynamic prompting of LLM-generated sections. Additional randomization was applied to dates, addresses, document metadata, and overall styling. While most narrative content such as clinical course and medical history is generated using the LLM, structured tables for lab results and medications are generated deterministically and appended to the letter. Based on an analysis of authentic documents, we identified 5 standard lab value section formats and 6 types of medication sections. These formats were hard-coded and selected randomly during generation to match observed documentation styles (Supplement D in Multimedia Appendix 1). An example of an anonymized and simplified synthetic discharge letter is shown in Figure 3 along with the intermediate JSON format.

This JSON format is designed to be populated based on the number of entities and hierarchical levels present in the discharge letter. With the exception of the introduction and tumor information sections, the remaining fields can accommodate multiple dictionaries, each of which has the potential to be postprocessed into an FHIR resource. In the figure, the medical entities to be extracted are highlighted in their respective hierarchy in the JSON.

### Model Training and Evaluation

The model was fine-tuned on the PIGEON dataset, which was split at the patient level into 90% training and 10% test sets. We used instruction-based fine-tuning, using prompts designed to extract key relevant medical entities and map them directly to their corresponding hierarchy in the intermediate JSON format. Training was conducted on a single NVIDIA A100 GPU, leveraging the Unsloth library [26] for memory-efficient fine-tuning. Additionally, mixed-precision training (Floating-Point 16/Bfloat16 [used for mixed-precision training]) [27] and gradient accumulation were implemented to optimize computational throughput and memory usage. Detailed hyperparameters are presented in Supplement C in Multimedia Appendix 1.

Furthermore, the model was evaluated for its capability to produce the correct intermediate JSON schema. The JSON output was flattened to compare the labels against the model extractions. Performance was quantified using the *F*_1_-score, the harmonic mean of precision and recall, which balances the trade-off between completeness and correctness of extraction. All medical code predictions (*International Classification of Diseases* [*ICD*], Anatomical Therapeutic Chemical [ATC], Operation and Procedure Classification [OPS]) were evaluated using Jaccard similarity, as these fields represent unordered sets of variable length where set-level agreement is more informative than positional matching [28], while remaining extractions with single expected values were evaluated through direct match, where exact correspondence is required. The model was evaluated using a synthetic and a real-world discharge letter test dataset in which predictions were reviewed by clinicians. The synthetic test set was used to assess the performance of the fine-tuned models on a large scale, whereas the real-world dataset evaluated the model’s capability on actual clinical discharge letters.

For both evaluation sets, we compare the model with generalist models with one-shot prompting. To our knowledge, no publicly available fine-tuned open-source model currently exists for multientity clinical information extraction from German discharge letters into FHIR-compatible structured formats, making a comparison with an adapted task-specific baseline infeasible at the time of this study. A few-shot approach was also not viable due to context window constraints: German discharge letters can be lengthy documents, and the target JSON schema is itself large. The one-shot prompts were iteratively optimized to maximize performance within these constraints (Supplement A in Multimedia Appendix 1).

To perform the human evaluation, a custom React-based web app was developed. The evaluation team included 3 experienced dermatology clinicians from University Hospital Essen and 1 medical student with 3 years of experience in medical text annotation. This group assessed outputs exclusively from the PIGEON and Qwen3 (235B) models, using a set of 30 discharge letters from various clinical departments. Scoring followed a case-level approach: each extracted clinical entity was treated as a composite case consisting of its constituent fields (eg, a diagnosis comprises the diagnosis name, the associated *ICD* code, and the date; a medication comprises the medication name, dosage, and ATC code; a laboratory value comprises the parameter name and its value). A case was marked as correct only if all constituent fields were accurately extracted; if any single field was incorrect or missing, the entire case was scored as wrong. This strict, all-or-nothing scoring ensures that the reported accuracy reflects clinically meaningful correctness rather than partial matches. For each instance, annotators verified the model’s response and manually provided the correct answer if the model was wrong. To assess interannotator reliability, 6 discharge letters were independently annotated by 2 dermatology annotators, yielding 187 comparable annotation pairs across all clinical domains. Given the high workload required for this detailed review, this study was designed as a proof-of-concept study that prioritizes the depth and clinical accuracy of the evaluation over a large-scale dataset.

### Code Prediction Enhancement

Additionally, a postprocessing module using a retrieval-augmented generation (RAG) [29] module to enhance *International Classification of Diseases, 10th Revision* (*ICD-10*), ATC, and OPS code prediction was implemented and evaluated. Each extracted entity display was postprocessed with a retrieval-augmented corrector to suppress code hallucinations. This enhancement system embeds the entire vocabulary of *ICD*, ATC, and OPS codes, sourced by merging official BfArM catalogs with institution-specific code-description mappings from our FHIR repository, using a multilingual sentence-transformer (paraphrase-multilingual-MiniLM-L12-v2) into a Facebook AI Similarity Search [30] index; retrieves the top-10 semantically closest code candidates for the entity name via cosine similarity; supplements them with authoritative code descriptions fetched from the official website (only for *ICD* correction) [31]; and then prompts a deterministic MedGemma 4B vLLM end point to pick the single most plausible code. The model is instructed to stay within the retrieved candidate set or the lookup vocabulary, and the chosen code replaces the raw generation in the final output JSON. When retrieval confidence is low (top-1 cosine similarity to the nearest code candidate below 0.9), the system falls back to the original model prediction to avoid introducing additional errors. All prompts used in the training, inference, and code prediction are provided in Supplement A in Multimedia Appendix 1.

## Results

### Cohort Characteristics

**Figure 4.**
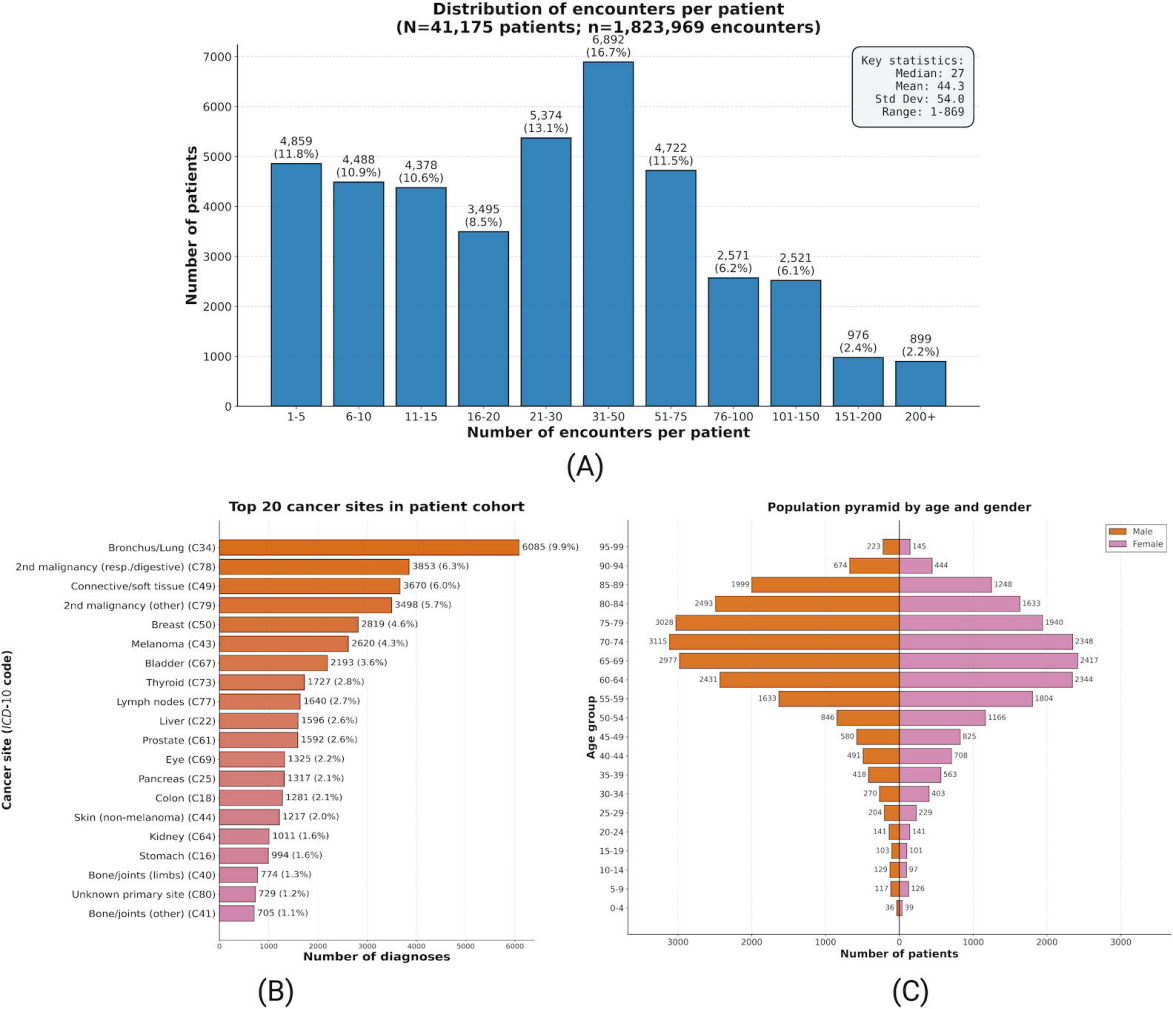
Overview of patient cohort demographics and clinical characteristics. (A) Top: distribution of health care use, measured by the range of medical encounters per patient. (B) Bottom left: the top 20 cancer sites within the cohort, ranked by the frequency of *International Classification of Diseases, 10th Revision* (*ICD-10*) code classifications. (C) Bottom right: population pyramid showing the distribution of patients by age and gender.

The PIGEON dataset comprised 41,175 patients (n=18,982, 46.1% female), with malignant neoplasms of the bronchi or lungs being the most prevalent diagnosis (*ICD-10* code C34, n=4076, 9.9%). Among those patients, encounter numbers varied across the cohort with a median number of encounters per patient of 27 (IQR 11‐56). While a substantial proportion (n=6876, 16.7%) of patients had between 31 and 50 encounters, given the chronic and complex nature of their conditions, primarily cancer, these patients typically have a protracted clinical journey spanning multiple years. This longitudinal course necessitates frequent clinical visits, each generating substantial volumes of FHIR resources, which are visualized in Figure 4. A comprehensive summary of the cohort’s baseline characteristics, including age distribution, diagnostic counts, and quantification of FHIR resources per patient, is provided in Table 2.

**Table 2.** Demographic and clinical characteristics of the patient cohort^a^ (N=41,175).

Category	Value
Female, n (%)	18,752 (46.1)
Male, n (%)	21,943 (53.9)
Age at last encounter in years, mean (SD)	66.3 (16.8)
Encounters per patient, median (IQR)	27.0 (11.0‐56.0)
FHIR^b^ resources per patient, median (IQR)	656 (190‐1941)
Main diagnoses per patient, median (IQR)	2 (1-3)
Patients with >1 main diagnosis, n (%)	34,068 (82.7)

a The table summarizes the baseline data for the study cohort, detailing patient demographics including age and gender distribution. It further provides quantitative metrics for clinical data volume.

b FHIR: Fast Healthcare Interoperability Resources.

To ensure comprehensive coverage of clinical scenarios and maximize the datasets’ generalizability, we analyzed the distribution of the main diagnoses. Figure 4B illustrates the localization of cancers among the patients. This distribution is based solely on the data available on the servers of the investigating site and is naturally influenced by regional cancer epidemiology. As depicted in Figure 4B, the dataset incorporates a broad spectrum of cancer cases. Furthermore, the distribution of encounters per patient, presented in Figure 4A, illustrates the variability in health care utilization and data density across the cohort. This factor influences the number of primary diagnoses per patient and helps understand the extent of concurrent clinical journeys a single patient has in the data repository.

To digitally represent these journeys, we used specific FHIR resources that serve as the foundational data units, encapsulating discrete medical information such as diagnoses, vital signs, medications, diagnostic reports, tumor information, and procedure details. Each patient journey in the study cohort included Patient, Encounter, Condition, Procedure, Observation, and MedicationStatement resources including detailed descriptions provided in Supplement G in Multimedia Appendix 1.

The collected FHIR data comprised 57 million resources, including 47 million Observations, 3.6 million Conditions, 2.1 million Procedures, and 646,000 MedicationStatements. It includes 10,000 unique *ICD* codes and 11,000 German OPS codes. This extensive and diverse data corpus provides a robust representation of a wide range of clinical scenarios, and the resulting dataset is designated as the FHIR cache.

### Synthetic Discharge Letter Evaluation

Table 3 reports PIGEON’s performance against 3 general-purpose baselines across all evaluation categories. The largest performance gaps appear in the medical-code extraction tasks (full *ICD*, OPS, ATC), where domain-specific knowledge is most critical.

**Table 3.** Benchmarking of the PIGEON^a^ model against state-of-the-art large language models (LLMs) across stratified clinical data domains^b^.

Evaluation category and metric			PIGEON	Qwen	LLaMA	OSS
Schema validity						
Valid JSON schema (%)			99.61	99.94	99.71	87.25
Diagnosis fields^c^						
*ICD*^d^ chapter			0.9557	0.7789	0.8078	0.7096
*ICD* category			0.8795	0.5969	0.5962	0.5464
*ICD* (full code)			0.8395	0.5003	0.4784	0.4517
Tumor information field						
Average tumor-related score			0.9912	0.8752	0.8494	0.7287
Free-text fields^c^						
Labs			0.9938	0.6750	0.6178	0.1429
Medications			0.9776	0.8859	0.8566	0.7776
Procedure codes			0.6345	0.1709	0.3550	0.0237
Procedures			0.8972	0.5158	0.5602	0.2954
Side diagnosis *ICD* chapter			0.8961	0.5641	0.4873	0.1482
Side diagnosis *ICD* category			0.8026	0.3563	0.3385	0.1240
Side diagnosis *ICD* (full code)			0.7516	0.2838	0.2685	0.1143
Body values			0.9938	0.8464	0.8460	0.8687
Other data fields^c^						
Introduction			0.9534	0.7261	0.7853	0.7897
Lab values			0.9990	0.9827	0.9583	0.8504
Medication ATC^e^ codes			0.9368	0.8292	0.7554	0.7495
Medication dosages			0.9987	0.9528	0.8877	0.8950
Medication names			0.9997	0.9373	0.8342	0.8580

a PIGEON: Patient Information Generation from Organized Notes.

b The PIGEON model was evaluated against 3 leading open-weights models: Qwen3 (235B), LLaMA 3.3 (70B), and GPT-OSS (120B). The results reflect raw model outputs; no postprocessing or external schema validation was applied. The scores represent the mean performance across 10 iterations of the test dataset. For all models, each inference run required approximately 1 hour to complete.

c Performance is reported via *F*_1_-scores.

d ICD: *International Classification of Diseases*.

e ATC: Anatomical Therapeutic Chemical.

The evaluation assesses PIGEON’s performance across various categories, including the particularly challenging domain of medical code extraction, where PIGEON substantially outperforms all baselines. As shown in Table 3, generalist baselines experience drastic performance drops in these rigorous tasks; for instance, Qwen3 achieves only an *F*_1_-score of 0.171 on procedure codes. In contrast, PIGEON maintains a strong macroaveraged *F*_1_-score of 0.912 across all 17 evaluated metrics. Even on the granular full-code *ICD* task, it achieves an *F*_1_-score of 0.840 (compared to Qwen3’s 0.500) and outperforms baselines on procedure codes with an *F*_1_-score of 0.635. Representative error examples illustrating the dominant failure modes of the baseline models (empty *ICD* and OPS codes, incorrect subdigits) are provided in Supplement E in Multimedia Appendix 1.

We present a quantitative evaluation in Table 4 demonstrating how the RAG module improves code correction and overall prediction accuracy.

**Table 4.** Quantitative impact of the RAG^a^ postprocessing module on medical entity prediction.^b^

Category	Before RAG	After RAG	Improvement
*ICD*^c^ chapter	0.9579	0.9540	–0.0039
*ICD* category	0.8795	0.9009	+0.0214
*ICD* (full code)	0.7314	0.8666	+0.1352
Procedure codes OPS^d^	0.6345	0.7295	+0.095
Medication ATC^e^ codes	0.8523	0.9178	+0.0655
Free-text side diagnosis *ICD* chapter	0.9077	0.9114	+0.0037
Free-text side diagnosis *ICD* category	0.8276	0.8400	+0.0124
Free-text side diagnosis *ICD* (full code)	0.6621	0.7972	+0.1351

a RAG: retrieval-augmented generation.

b The table compares *F*_1_-scores across diagnostic (*International Classification of Diseases, 10th Revision*), procedural (Operation and Procedure Classification), and pharmaceutical (Anatomical Therapeutic Chemical) domains before and after the application of the retrieval-based corrector.

c *ICD*: *International Classification of Diseases*.

d OPS: Operation and Procedure Code.

e ATC: Anatomical Therapeutic Chemical.

The RAG postprocessing module demonstrates clear effectiveness in correcting granular predictions, yielding F1 improvements of roughly 13.5 percentage points for full *ICD* codes and 9.5 percentage points for OPS procedures. Consequently, the system achieves significantly higher scores in assigning specific medical codes.

### Human Evaluation

The human-in-the-loop evaluation was conducted on 30 real-world discharge letters from various clinical departments. The quantitative results, summarized in Table 5, demonstrate that the PIGEON model achieved superior fidelity across most fields, recording an average accuracy of 87.5% (SD 10.6%) compared to 72.2% (SD 21.6%) for the general-purpose Qwen3-235B model.

**Table 5.** Quantitative results of the human-in-the-loop validation.^a^

Category	PIGEON^b^ model			Qwen3-235B		
	Correct (%)	Incorrect (%)	Letters, n	Correct (%)	Incorrect (%)	Letters, n
Patient information	99.7	0.3	297	99.6	0.4	282
Main Diagnosis (Category)	78.9	21.1	237	75.8	24.2	194
Main Diagnosis (Chapter)	86.0	14.0	236	81.4	18.6	194
Free Text Diagnosis (Category)	80.1	19.9	156	53.3	46.7	167
Free Text Diagnosis (Chapter)	83.8	16.2	154	56.3	43.7	167
Medications	93.0	7.0	171	79.7	20.3	197
Free Text Medications	89.5	10.5	19	26.8	73.2	97
Laboratory Values	97.9	2.1	327	96.9	3.1	323
Free Text Laboratory Values	95.5	4.5	110	43.4	56.6	53
Procedures	61.3	38.7	137	43.5	56.5	138
Tumor Information	86.1	13.9	79	56.5	43.5	223
Average	87.5	12.5	1923	72.2	27.8	2035

a The table compares the accuracy of the PIGEON (Patient Information Generation from Organized Notes) model and Qwen3-235B when evaluated against an expert-verified reference dataset of 30 manually annotated discharge letters. Performance is categorized by clinical domain. The predictions include postprocessing with the retrieval-augmented generation corrector module.

b PIGEON: Patient Information Generation from Organized Notes.

The largest differences in Table 5 between PIGEON and Qwen3 appear in the free-text categories (Free Text Diagnoses, Free Text Medications, Free Text Lab Values), while procedures remain the weakest category for both systems. While the Qwen3-235B model demonstrated high performance given the complexity of the task, successfully identifying correct codes with the assistance of the RAG corrector, it frequently struggled with structural coherence. The evaluation revealed that Qwen3-235B often repeated information, misallocated data within the hierarchy, or appended irrelevant details to value fields. Hence, it produced more information than the PIGEON model, especially in tumor information and free-text medications. These structural inconsistencies impacted its performance in unstructured categories, most notably in Free Text Medications and Free Text Laboratory Values, where precise semantic mapping is critical. In contrast, qualitative feedback from human annotators highlighted the PIGEON model’s reliability and precision. The PIGEON model exhibited negligible hallucination, adopting a conservative extraction strategy where it omitted fields rather than generating plausible but incorrect data when confidence was low. A detailed hierarchy-aware error analysis of the residual procedure errors addressing the OPS-catalog ambiguity is provided in Supplement F in Multimedia Appendix 1.

To assess the consistency of the human evaluation, interannotator agreement was calculated on the subset of 6 discharge letters that were independently reviewed by 2 experienced dermatologist annotators. Across 187 comparable annotation pairs (comprising entity-level judgments across all clinical domains), the annotators reached agreement on 164 (87.7%) cases and disagreed on 23 (12.3%) cases. Cohen κ [32] was 0.751, which falls within the “substantial agreement” range according to Landis and Koch (0.61‐0.80) [33].

## Discussion

### Principal Findings

We propose a framework intended to help mitigate bottlenecks in health care. By generating synthetic training data from a live FHIR server, this approach could serve as a reference for other institutions building secure, on-premise NLP solutions.

Through the use of the PIGEON dataset, created using the random sample generator, the fine-tuned PIGEON model achieved a competitive score on the FHIR resource generation task. The inclusion of popular open-source LLMs in the evaluation further underscored the efficacy of fine-tuned open-source models for downstream tasks. The PIGEON model achieved better performance than larger general-purpose models while offering the flexibility and cost-effectiveness associated with lower-parameter solutions, a finding consistent with recent work on resource-constrained clinical extraction [34]. This finding aligns with the growing paradigm of small language models, where recent benchmarks indicate that compact, domain-specialized models can match the reasoning capabilities of massive generalist models in clinical settings while enabling efficient edge deployment [35,36].

In the context of related work, recent studies suggest that LLMs offer advantages over traditional NLP techniques for clinical information extraction [34,37-39]. Within this domain, agentic workflows have emerged as a promising solution for complex reasoning tasks in health care [17,40]. However, comparative analysis is often constrained by the predominance of commercial, closed-source LLMs and heterogeneous evaluation metrics. While works using proprietary models (eg, GPT-4) demonstrate high efficacy, challenges persist regarding data governance and General Data Protection Regulation adherence [41,42]. This study differentiates itself from related work through its approach to dataset creation using real FHIR resources and by fine-tuning a resource-effective LLM. This approach allows for feasible on-premise implementation on clinical infrastructure where data privacy is critical, addressing the “closed versus open” deployment dilemma highlighted in recent regulatory frameworks [41].

A distinct advantage of this work is its capability to process unstructured clinical text. While standard extract, transform, load (ETL) pipelines remain essential for managing structured data, our approach serves as a complementary extension for handling complex clinical narratives. This supports a future trajectory where robust ETL infrastructure and flexible LLM-driven solutions coexist to address the full spectrum of health care data. In a daily clinical routine, this framework is intended to operate as a background service that parses discharge letters and populates the EHR with pre-extracted information for clinician verification, validating recent hypotheses that LLMs can effectively automate unstructured-to-structured data conversion [43,44]. Although our quantitative evaluation used deterministic coding accuracy as a deliberately strict benchmark, clinical workflows rarely require physicians to verify granular medical codes directly. In most health care systems, *ICD* coding is performed by dedicated professional coders rather than treating physicians [45]. What clinicians need at the point of care is the correct identification and display of clinical entities: diagnosis names, medication names, laboratory values, and procedure descriptions. Qualitative feedback from our physician annotators consistently indicated that when the model identifies a clinical entity, it provides a plausible and contextually appropriate display name while reliably avoiding negated or excluded findings. This suggests that PIGEON shows promise as a pragmatic, low-cost information extractor within routine clinical workflows. By surfacing structured clinical summaries for physician review, the framework could shift the clinician’s role from manual data entry to verification of preextracted information. Combined with the structured output constraints and retrieval-grounded postprocessing validated in the *Results* section, this design supports output-level verifiability and maintains human-in-the-loop oversight, which will remain essential as the system progresses toward routine clinical use. Furthermore, the modular framework architecture comprising the fine-tuned extraction model, the RAG-based code corrector, and preprocessing modules allows each component to be independently improved, so that future enhancements could translate into gains at the point of care without requiring retraining of the entire system.

To operationalize this workflow, we developed a comprehensive clinical application integrating PIGEON, which is being prepared for a controlled pilot deployment in the Department of Dermatology at the University Hospital Essen. This implementation demonstrates the end-to-end framework under realistic operating conditions, including connections to preprocessing modules, such as Optical Character Recognition, to handle scanned clinical documents. A detailed explanation of this application’s architecture and functionality is provided in Supplement B in Multimedia Appendix 1.

Compared to existing approaches for clinical information extraction, the synthetic-data fine-tuning paradigm introduced here offers distinct advantages in scalability and generalization. Traditional supervised methods depend on costly manual annotation of real clinical documents, which limits dataset size and introduces annotator bias. Rule-based and named entity recognition pipelines, while reliable for narrow extraction tasks, require extensive domain-specific engineering and do not generalize well across clinical settings or documentation styles. Proprietary LLM-based solutions can achieve strong zero-shot performance but are constrained by data governance requirements that prohibit the transfer of patient data to external services. In contrast, the PIGEON framework generates training data programmatically from structured FHIR resources already available in the institutional backend, eliminating the need for manual annotation entirely. Because the random sample generator operates on the FHIR cache, any institution that adopts the FHIR standard could in principle replicate this workflow with its own data, producing a fine-tuned model tailored to its local documentation patterns, including department-specific terminology and formatting conventions without sharing patient data externally. The synthetic-data evaluation, which spans discharge letters from various clinical departments, suggests that the approach is not specialty-bound, although this finding requires confirmation on real-world data from independent sites. Taken together, these results establish the technical feasibility of the approach; broader clinical adoption will require prospective multisite validation and formal regulatory assessment before routine use.

The study is subject to several limitations that constrain current conclusions. All evaluations were conducted at a single site, the University Hospital Essen, whose productive FHIR R4 server contains over 2 billion resources and is, to our knowledge, one of the largest single-institution FHIR repositories in Europe. While this scale supports a robust within-site evaluation, it does not establish that performance generalizes to hospitals with different documentation styles, FHIR profiles, or coding conventions. Cross-institutional validation was beyond the scope of this study under General Data Protection Regulation and our ethics approval (24‐12111-BO). The framework was also developed exclusively for German clinical documents, which restricts immediate generalizability to other languages. The real-world clinical evaluation was based on 30 discharge letters reviewed by clinicians from a single institution. Although the case-level scoring methodology was conservative and clinician collaboration ensured high data quality, this sample represents a narrow slice of documentation diversity, and broader multidepartmental validation would be required before routine clinical deployment. Procedure code extraction is the weakest domain in our evaluation and the most clinically relevant limitation for practical use. OPS codes are inherently difficult to extract: they encode anatomical site, laterality, technique, and access route in a single granular identifier and frequently require contextual inference from scattered narrative descriptions. Our hierarchy-aware analysis (Supplement F in Multimedia Appendix 1) indicates that the errors are concentrated at well-recognized points of OPS-catalog ambiguity [46] rather than reflecting random miscoding. A further, inherent limitation arises from our random synthetic-letter generation, whose training OPS distribution is shaped by the source FHIR repository rather than by the real-world oncology deployment cohort. In practical terms, procedure-related output should be treated as a structured draft that supports rather than replaces expert coding. On the modeling side, our experiments were restricted to fine-tuning a single LLM, leaving open whether alternative architectures or ensemble strategies could yield further improvements. The comparison against alternative systems is restricted to one-shot prompting of general-purpose open-source LLMs (Qwen3, LLaMA, GPT-OSS) and does not include a fine-tuned or task-specific baseline. No publicly available task-specific system for German clinical information extraction to FHIR existed at the time of this study, and as detailed in the *Methods* section, fine-tuning the baseline models on the same PIGEON dataset would have conflated framework contribution with architectural differences. The observed performance gap should therefore be interpreted as reflecting the advantage of domain-specific fine-tuning over prompt-based extraction under realistic deployment conditions, rather than as a claim of architectural superiority over alternative task-specific systems.

Looking ahead, the modular design of this framework offers potential for further extension, particularly when considering the rapid trajectory of LLM capabilities. While this study focused on German clinical documents, the methodology is inherently flexible; future work could extend this framework to multilingual datasets to facilitate cross-border interoperability [23], while also upgrading the random sample generator itself. By leveraging more advanced, reasoning-capable LLMs as teacher models, the framework could move beyond structural randomization to synthesize complex, high-fidelity patient histories. This evolution would allow institutions to generate synthetic training data for edge cases, effectively solving the “long-tail” data scarcity problem without the privacy risks associated with real-world records. To mitigate the bottleneck of manual annotation, we suggest using an LLM-as-a-judge framework. Recent studies [47,48] suggest that stronger, reasoning-heavy models can serve as scalable automated evaluators for clinical NLP tasks, significantly expanding the validation set without incurring additional human cost. Addressing the remaining constraints by incorporating multilingual datasets represents a valuable direction for future research. In parallel with multilingual extension, prospective multisite validation across other German university hospitals is the most immediate next step. The German Medizininformatik-Initiative [9] and its associated FHIR-aligned data integration centers provide a natural infrastructural basis for such a study, since participating sites already operate FHIR R4 backends with comparable resource profiles. This allows the random sample generator and fine-tuning workflow to be reinstantiated on each site’s own data without transferring patient records, directly testing the framework’s portability hypothesis under realistic data-governance constraints. Future investigations should also focus on combining this generative approach with multiple small language models by dividing the task into smaller subtasks to enhance computational efficiency. Furthermore, procedure code extraction would benefit from targeted training on large collections of real-world procedure descriptions paired with verified OPS codes and complemented by catalog-aware postprocessing that handles common multichapter ambiguity classes permissively. The inherent ambiguity of procedure-to-code mappings will, however, continue to pose challenges and motivates the continued use of expert review in the procedure-extraction workflow. Finally, as task-specific baselines for German clinical information extraction become available, benchmarking against such adapted systems would provide a more rigorous evaluation of the proposed architecture.

### Conclusions

This study presents and evaluates a privacy-preserving and resource-efficient framework for enhancing health care data interoperability. We demonstrate that a resource-efficient, open-source LLM, fine-tuned on a synthetic dataset derived from authentic FHIR resources, can transform unstructured German discharge letters into standardized formats with competitive accuracy. This approach complements traditional ETL pipelines and offers a secure alternative to large, proprietary models. As a single-site proof-of-concept conducted on one of Europe’s largest institutional FHIR repositories, this work demonstrates the technical feasibility of the approach and provides a practical foundation for health care systems seeking to leverage unstructured clinical data. Prospective multisite validation across independent FHIR-aligned institutions remains a necessary next step before routine clinical use, with the long-term goal of improving operational efficiency and patient care.

## Supplementary material

10.2196/92413 Multimedia Appendix 1 Prompt and synthetic data generation templates along with training parameters and error analyses.

## Abbreviations:

ATC: Anatomical Therapeutic Chemical
EHR: electronic health record
ETL: extract, transform, load
FHIR: Fast Healthcare Interoperability Resources
*ICD-10*: *International Classification of Diseases, 10th Revision*
LLM: large language model
NLP: natural language processing
OPS: Operation and Procedure Classification
PIGEON: Patient Information Generation from Organized Notes
RAG: retrieval-augmented generation
