# Supplementary material for: Extracting Medical Information From Unstructured Clinical Text Using Large Language Models to Enhance Health Care Interoperability: Proof-of-Concept Study
Source: J Med Internet Res. 2026 Jul 2;28:e92413. doi: 10.2196/92413 (PMC13325620; doi:10.2196/92413)
Supplement: Multimedia Appendix 1 [file jmir-v28-e92413-s001.docx]

# Supplementary Material

## A Used Prompts

All the used prompts are given below translated from German to English. The prompts are never static and dynamic parts include the sensitive patient data.

### A.1 Prompts for Anamnese and Epikrise:

| Generate a coherent and medically relevant 'Anamnesis' section for a patient. Use the provided structured data and examples of real anamneses for this purpose.  Use the following structured data of the patient. Integrate this information naturally into the report. Ensure that information regarding known and excluded diagnoses as well as current and not prescribed medication (names only) is processed correctly.  {structured_data_heading}{structured_data_block}  Here are three examples of real anamneses. Use these examples to understand the typical style, phrasing, and the manner in which medically relevant routine information or "filler texts" are incorporated, which help to tell a story about the medical history and the current condition of the patient. Adopt similar narrative elements while integrating the structured data mentioned above.  Example 1:  ---  {anamnese_example_1}  ---  Example 2:  ---  {anamnese_example_2}  ---  Example 3:  ---  {anamnese_example_3}  ---  Create the Anamnesis section. This should be fluently readable, naturally integrate the provided structured information, and contain narrative elements similar to the examples.  Return the output in JSON format. The JSON object MUST contain the following keys:  1. `anamnese_section`: The complete text of the Anamnesis section.  The indices must match the generated text exactly. Return a separate entry with the exact positions for every aspect (e.g., lab names and lab value, body weight and body height).  Example for the JSON format:  {{  "anamnese_section": "Patient was admitted with diabetes mellitus. Metformin was prescribed. The body weight is 80 kg. The body height is 175 cm. Lab: HbA1c 7.2%. A 3D-CT scan was performed.",  }}  Ensure that the indices are correct and consistent with the generated text. Output NO further explanations or comments, but only the JSON object. |
| --- |

### A.2 Prompts for Finetuning:

| Extract medical information from the following medical text and structure it as JSON.  RULES:  - Answer ONLY as a valid JSON object  - Extract only information explicitly mentioned in the text  - Indicate missing information as empty values ("", [], {{}})  - Use complete JSON structure according to schema  JSON-SCHEMA:  {{  "introduction": {{  "family_name": "",  "given_name": "",  "birth_date": "",  "gender": "",  "address_street": "",  "address_city": "",  "address_postal_code": "",  "stationary_type": "",  "encounter_start_date": "",  "encounter_end_date": ""  }},  "diagnoses": [  {{  "type": "main_diagnosis",  "name": "",  "icd10gm_code": "",  "date": ""  }},  {{  "type": "side_diagnosis",  "name": "",  "icd10gm_code": "",  "date": ""  }}  ],  "tumor_informations": [  {{  "type": "pathological",  "stage": "",  "t": "",  "n": "",  "m": "",  "date": ""  }},  {{  "type": "clinical",  "t": "",  "n": "",  "m": "",  "date": ""  }},  {{  "type": "histology",  "histology": "",  "date": ""  }},  {{  "type": "overall_status",  "status_de": "",  "date": ""  }},  {{  "type": "progression",  "description_de": "",  "date": ""  }},  {{  "type": "tumor_marker",  "marker": "",  "value": 0.0,  "unit": "",  "date": ""  }},  {{  "type": "smoking_status",  "status": "",  "date": ""  }},  {{  "type": "ecog_performance",  "score": 0,  "date": ""  }},  {{  "type": "comorbidities",  "conditions": [],  "date": ""  }},  {{  "type": "operations",  "procedures": [],  "date": ""  }},  {{  "type": "radiotherapy",  "procedures": [],  "date": ""  }}  ],  "medication": [],  "lab_values": [  {{  "lab_name": "",  "lab_value": 0.0  }}  ],  "free_text": {{  "lab_values": [  {{  "name": "",  "value": ""  }}  ],  "medications": [],  "body_values": [],  "procedures": [  {{  "procedure_name": "",  "procedure_code": "",  "code_type": ""  }}  ],  "diagnoses": [  {{  "type": "side_diagnosis",  "official_name": "",  "icd10gm_code": ""  }}  ]  }}  }}  MEDICAL TEXT:  {medical_text} |
| --- |

### A.3 Example RAG Based Code Extraction Prompt

| You are a medical expert in ICD-10 coding. Select the correct ICD-10 code for the given diagnosis.  DIAGNOSIS: {diagnosis_name}  {original_text}  ALTERNATIVE CANDIDATES:  {candidates_text}  IMPORTANT:  - Respond ONLY with the ICD-10 code (Format: Letter + 2 digits + optional .digits)  - Examples: A01.0, C44.5, I25.1  - The OFFICIAL DESCRIPTION is the authoritative definition of the code  - Ensure that if no localization is recognizable, you choose the more general code (usually unspecified)  - You can keep the ORIGINAL CODE if it is already correct  - No explanations, no additional words, just the code!  Code: |
| --- |

### A.4 Prompt for One-Shot Qwen3

The output schema is visible in the prompt in the example answer part.

| Extract medical information from the following medical text and structure it as JSON.  RULES:  - Response ONLY as a valid JSON object  - Extract only information explicitly mentioned in the text  - Return missing information as empty values ("", [], {})  - Use the complete JSON structure according to the schema  - Use the official ICD-10-GM codes for diagnoses, ATC codes for medications, and OPS codes for procedures.  JSON-SCHEMA:  {{  "introduction": {{  "family_name": "",  "given_name": "",  "birth_date": "",  "gender": "",  "address_street": "",  "address_city": "",  "address_postal_code": "",  "stationary_type": "",  "encounter_start_date": "",  "encounter_end_date": ""  }},  "diagnoses": [  {{  "type": "main_diagnosis",  "name": "",  "icd10gm_code": "",  "date": ""  }},  {{  "type": "side_diagnosis",  "name": "",  "icd10gm_code": "",  "date": ""  }}  ],  "tumor_informations": [  {{  "type": "pathological",  "stage": "",  "t": "",  "n": "",  "m": "",  "date": ""  }},  {{  "type": "clinical",  "t": "",  "n": "",  "m": "",  "date": ""  }},  {{  "type": "histology",  "histology": "",  "date": ""  }},  {{  "type": "overall_status",  "status_de": "",  "date": ""  }},  {{  "type": "progression",  "description_de": "",  "date": ""  }},  {{  "type": "tumor_marker",  "marker": "",  "value": 0.0,  "unit": "",  "date": ""  }},  {{  "type": "smoking_status",  "status": "",  "date": ""  }},  {{  "type": "ecog_performance",  "score": "",  "date": ""  }},  {{  "type": "comorbidities",  "conditions": [],  "date": ""  }},  {{  "type": "operations",  "procedures": [],  "date": ""  }},  {{  "type": "radiotherapy",  "procedures": [],  "date": ""  }}  ],  "medication": [],  "lab_values": [  {{  "lab_name": "",  "lab_value": 0.0  }}  ],  "free_text": {{  "lab_values": [  {{  "name": "",  "value": ""  }}  ],  "medications": [],  "body_values": [],  "procedures": [  {{  "procedure_name": "",  "ops_code": "",  "code_type": ""  }}  ],  "diagnoses": [  {{  "type": "side_diagnosis",  "official_name": "",  "icd10gm_code": ""  }}  ]  }}  }}  ONE SHOT EXAMPLE:  Dear Colleagues,  Patient John Doe, born 06.08.1956, was treated as an inpatient from 20.05.2018 to 24.05.2018.  Diagnoses:  Malignant adenoma of the pulmonary lower lobe  Round focal lesion of the lung with suspicion of granulomatosis (Diagnosis Date: 10.2024)  Affective dysregulation (known since 02/2025)  Obstructive lung disease with preserved lung function (FEV1 ≥ 50%)  Chronic bronchitis with mild airflow limitation (2025)  Major depressive disorder  Chronic nicotine abuse  Prostate adenoma  Chronic Obstructive Pulmonary Disease (COPD) (from 02.2025)  Moderate recurrent depressive disorder  Tumor Status:  Current stage (pTNM): T1a N0  Clinical cTNM: T2b N1 M0  Grading: poorly differentiated  Histology: Squamous cell carcinoma NOS  Overall tumor status: Complete remission with residual abnormalities (CRr)  Course / Progression: no tumor detectable  Tumor marker: LDH - 230 U/l  Smoking status: 60  ECOG Performance Status: Restriction in physical exertion, but ambulatory; able to carry out work of a light or sedentary nature (e.g., light house work, office work)  Relevant comorbidities: Chronic lung disease; Chronic Lung Disorder; Chronic lung disease  Anamnesis (History)  The patient is a single man who has been suffering from increasing dyspnea on exertion for several weeks. He reports a productive cough with mucopurulent sputum, occasional fever, as well as weight loss and night sweats. In the past, he was diagnosed with emphysematous COPD without long-term therapy. Active tuberculosis or other infectious causes could not be excluded so far. Furthermore, a sigmoid diverticulum and first-degree hemorrhoids are known. Additionally, there is a tobacco dependence syndrome with a long history of smoking. The patient has recently been permanently fitted with a small-bore pleural indwelling catheter, which was inserted therapeutically due to recurrent pleural effusion. The application for classification into a nursing grade was successful; the patient is currently dependent on home care. He is currently taking Quetiapine-1A Pharma 50 mg and Vitagamma Vitamin D3 1000 I.U. Systemic antibiotic therapy or other immunosuppressive medication is not currently prescribed. Laboratory chemistry shows an increased monocyte count of 16.0%, indicating a possible chronic inflammatory process.  Epicrisis (Discharge Summary)  The inpatient treatment of Mr. N. was carried out for differential diagnosis and intensive medical clarification of complex pulmonary and cardiac symptoms, taking into account multiple morbidities. Upon admission, there was an exacerbated COPD with a relevant FEV1 decline to 35-50% of the predicted value with an underlying infectious origin. In addition, there was a pyothorax development with parenchymal lung involvement and fistula formation, which required close antibiotic therapy and pulmonological intervention. Concurrently, a filariasis screening examination was performed to exclude a possible parasitic component. Attacks of tachycardia as well as a paroxysmal anxiety syndrome contributed to the clinical complexity and required an interdisciplinary approach.  During the inpatient stay, several relevant diagnostic procedures were performed, including gene expression analyses on solid malignant neoplasms with analysis of 1 to 2 target structures as well as high-throughput sequencing (NGS) for detailed characterization of genetic alterations considering coding and regulatory sequences. In addition, a whole-body scintigraphy was performed for localization diagnostics.  At discharge, clinical stabilization was evident with unremarkable courses of the relevant laboratory parameters. Eosinophil granulocytes were at 0.9% (absolute 0.04 x10⁹/L), the lymphocyte count was 1.56 x10⁹/L in the lower normal range. The recommended discharge medication with Trazaxiro 100 mg was initiated to accompany the psychosomatic symptoms and reduce possible anxiety attacks.  Following inpatient discharge, close specialist follow-up is recommended, particularly pulmonological and cardiological, supplemented by regular monitoring of genetic findings as part of oncological monitoring. The results of the performed molecular biological analyses will be communicated promptly and discussed in the interdisciplinary consultation.  Long-term Medication  Trade Name  Active Ingredient  Strength  Form  Morning  Noon  Evening  Night  Notes  Reason  Novaminsulfon Lichtenstein 500mg  2  2  2  2  Terablock 5mg  1  0  0  0  Eliquis 5mg  1  0  1  0  Novaminsulfon 500-1A Pharma  2  2  2  2  EMEND 80mg  1  0  0  0  MCP STADA 10mg  1  1  1  0  MOVICOL ready-to-drink 25ml sachet  1  1  1  0  Lab Parameters:  Test  Value  Flag  Status  Prev. Value  dated  Unit  Ref. Range  Baso.Granulocytes#  0.02  /nl  0.0 - 0.1  Baso. Granulocytes%  0.5  %  0.0 - 1.1  Leukocytes  4.26  /nl  3.6 - 9.2  Erythrocytes  4.04  L  /pl  4.5 - 5.6  Hemoglobin  12.4  g/dl  11.8 - 17.5  Hematocrit  0.371  L  l/l  0.4 - 0.5  MCV  91.8  fl  83.0 - 98.0  MCH  30.7  pg  28.0 - 33.0  MCHC  33.4  g/dl  32.0 - 36.0  Platelets  195.0  /nl  140.0 - 320.0  EXAMPLE ANSWER:  {  "introduction": {  "family_name": "Doe",  "given_name": "John",  "birth_date": "06.08.1956",  "gender": "male",  "address_street": "",  "address_city": "",  "address_postal_code": "",  "stationary_type": "inpatient",  "encounter_start_date": "20.05.2018",  "encounter_end_date": "24.05.2018"  },  "diagnoses": [  {  "type": "main_diagnosis",  "name": "Adenocarcinoma lower lobe",  "icd10gm_code": "C34.3",  "date": ""  },  {  "type": "main_diagnosis",  "name": "Round focus lung (susp. granulomatosis)",  "icd10gm_code": "D38.1",  "date": "10.2024"  },  {  "type": "side_diagnosis",  "name": "Sad mood",  "icd10gm_code": "F32.9",  "date": "02/2025"  },  {  "type": "side_diagnosis",  "name": "Other specified chronic obstructive pulmonary disease: FEV1 >= 50 % of predicted value",  "icd10gm_code": "J44.82",  "date": ""  },  {  "type": "side_diagnosis",  "name": "COPD I",  "icd10gm_code": "J44.83",  "date": "2025"  },  {  "type": "side_diagnosis",  "name": "Nicotine abuse",  "icd10gm_code": "F17.1",  "date": ""  },  {  "type": "side_diagnosis",  "name": "Prostatic hypertrophy",  "icd10gm_code": "N40",  "date": ""  },  {  "type": "side_diagnosis",  "name": "Chronic lung disease",  "icd10gm_code": "J44.9",  "date": "02.2025"  },  {  "type": "side_diagnosis",  "name": "Recurrent depressive disorder, moderate episode",  "icd10gm_code": "F33.1",  "date": ""  }  ],  "tumor_informations": [  {  "type": "pathological",  "stage": "",  "t": "T1a",  "n": "N0",  "m": "",  "date": ""  },  {  "type": "clinical",  "t": "T2b",  "n": "N1",  "m": "M0",  "date": ""  },  {  "type": "histology",  "histology": "Squamous cell carcinoma NOS",  "date": ""  },  {  "type": "overall_status",  "status_de": "Complete remission with residual abnormalities (CRr)",  "date": ""  },  {  "type": "progression",  "description_de": "no tumor detectable",  "date": ""  },  {  "type": "tumor_marker",  "marker": "LDH",  "value": 230,  "unit": "U/l",  "date": ""  },  {  "type": "smoking_status",  "status": 60,  "date": ""  },  {  "type": "ecog_performance",  "score": "Restriction in physical exertion, but ambulatory; able to carry out work of a light or sedentary nature (e.g., light house work, office work)",  "date": ""  },  {  "type": "comorbidities",  "conditions": [  "Chronic lung disease",  "Chronic Lung Disorder",  "Chronic lung disease"  ],  "date": ""  }  ],  "medication": [  {  "medication_name": "Novaminsulfon Lichtenstein 500mg",  "dosage_info": {  "Morning": 2.0,  "Noon": 2.0,  "Evening": 2.0,  "Night": 2.0  },  "atc_code": "N02BB02"  },  {  "medication_name": "Terablock 5mg",  "dosage_info": {  "Morning": 1.0,  "Noon": 0,  "Evening": 0,  "Night": 0  },  "atc_code": "G04CA03"  },  {  "medication_name": "Eliquis 5mg",  "dosage_info": {  "Morning": 1.0,  "Noon": 0,  "Evening": 1.0,  "Night": 0  },  "atc_code": "B01AF02"  },  {  "medication_name": "Novaminsulfon 500-1A Pharma",  "dosage_info": {  "Morning": 2.0,  "Noon": 2.0,  "Evening": 2.0,  "Night": 2.0  },  "atc_code": "N02BB02"  },  {  "medication_name": "EMEND 80mg",  "dosage_info": {  "Morning": 1.0,  "Noon": 0,  "Evening": 0,  "Night": 0  }  },  {  "medication_name": "MCP STADA 10mg",  "dosage_info": {  "Morning": 1.0,  "Noon": 1.0,  "Evening": 1.0,  "Night": 0  },  "atc_code": "A03FA01"  },  {  "medication_name": "MOVICOL ready-to-drink 25ml sachet",  "dosage_info": {  "Morning": 1.0,  "Noon": 1.0,  "Evening": 1.0,  "Night": 0  },  "atc_code": "A06AD65"  }  ],  "lab_values": [  {  "lab_name": "Baso.Granulocytes#",  "lab_value": 0.02  },  {  "lab_name": "Baso. Granulocytes%",  "lab_value": 0.5  },  {  "lab_name": "Leukocytes",  "lab_value": 4.26  },  {  "lab_name": "Erythrocytes",  "lab_value": 4.04  },  {  "lab_name": "Hemoglobin",  "lab_value": 12.4  },  {  "lab_name": "Hematocrit",  "lab_value": 0.371  },  {  "lab_name": "MCV",  "lab_value": 91.8  },  {  "lab_name": "MCH",  "lab_value": 30.7  },  {  "lab_name": "MCHC",  "lab_value": 33.4  },  {  "lab_name": "Platelets",  "lab_value": 195.0  }  ],  "free_text": {  "medications": [  {  "name": "Quetiapine-1A Pharma 50mg",  "dosage": "19:00\|1\|Tab.",  "atc_code": "N05AH04"  },  {  "name": "Vitagamma Vitamin D3 1000 I.E.",  "dosage": "07:00\|1\|Tab.",  "atc_code": "A11CC05"  },  {  "name": "Trazaxiro 100mg",  "dosage": "19:00\|1\|Tab."  }  ],  "procedures": [  {  "procedure_name": "Therapeutic drainage of the pleural cavity: Small-bore, permanent indwelling system",  "ops_code": "8-144.1",  "code_type": "ops"  },  {  "procedure_name": "Need for care: Successful application for classification into a nursing grade",  "ops_code": "9-984.b",  "code_type": "ops"  },  {  "procedure_name": "Performance of gene expression analyses in solid malignant neoplasms: Analysis of 1 to 2 target structures",  "ops_code": "1-992.0",  "code_type": "ops"  },  {  "procedure_name": "High-throughput sequencing [NGS] for analysis of genetic alterations in solid malignant neoplasms: Analysis of coding sequence including associated regulatory sequences, tissue sample: up to 5 kilobases",  "ops_code": "1-996.00",  "code_type": "ops"  },  {  "procedure_name": "Whole-body scintigraphy for localization diagnostics: Other",  "ops_code": "3-70c.x",  "code_type": "ops"  }  ],  "diagnoses": [  {  "type": "side_diagnosis",  "official_name": "Emphysematous COPD: without maintenance medication {COPD}",  "icd10gm_code": "J44.89"  },  {  "type": "side_diagnosis",  "official_name": "Hemorrhoids stage 1",  "icd10gm_code": "K64.0"  },  {  "type": "side_diagnosis",  "official_name": "Sigmoid diverticulum",  "icd10gm_code": "K57.30"  },  {  "type": "side_diagnosis",  "official_name": "History of chronic nicotine abuse",  "icd10gm_code": "F17.2"  },  {  "type": "side_diagnosis",  "official_name": "Screening Filariasis",  "icd10gm_code": "Z11"  },  {  "type": "side_diagnosis",  "official_name": "Pyothorax with fistula of lung parenchyma",  "icd10gm_code": "J86.00"  },  {  "type": "side_diagnosis",  "official_name": "Tachycardia NOS",  "icd10gm_code": "R00.0"  },  {  "type": "side_diagnosis",  "official_name": "Infectiously exacerbated COPD with FEV1 >= 35 % and < 50 % predicted value",  "icd10gm_code": "J44.02"  },  {  "type": "side_diagnosis",  "official_name": "Panic disorder (episodic paroxysmal anxiety)",  "icd10gm_code": "F41.0"  }  ],  "lab_values": [  {  "name": "Monocytes%",  "value": "16.0 (high)"  },  {  "name": "Eos. Granulocytes%",  "value": "0.9"  },  {  "name": "Eos. Granulocytes#",  "value": "0.04"  },  {  "name": "Lymphocytes#",  "value": "1.56"  }  ],  "body_values": []  }  }  Now extract the information from the following medical text and return ONLY the JSON object:  MEDICAL TEXT:  {medical_text} |
| --- |

Prompt for Qwen3 model includes one sample from synthetic dataset therefore it is anonymized.

##

##

##

## B Use Case for PIGEON: Prototype Application & Clinical Workflow

### B.1 Clinical Implementation

To demonstrate the practical utility of the fine-tuned model in a clinical setting, we developed a prototype application designed to streamline the ingestion of unstructured external medical documents. The application employs a multi-stage technical architecture capable of handling diverse input formats, such as PDFs and scanned images, to ensure high-fidelity data extraction. The ingestion process begins with Docling, which parses incoming files and checks for an existing text layer. If no text is detected, the system utilizes a hybrid strategy where a Vision Language Model (VLM) performs optical character recognition and layout analysis to recover the raw text. This text is then processed by our fine-tuned PIGEON model, which extracts entities into a defined JSON schema covering demographics, diagnoses, and tumor status. Subsequently, a Retrieval-Augmented Generation (RAG) module post-processes the output to validate terminology, specifically mapping diagnoses to valid ICD codes and procedures to OPS codes. Finally, the validated data is automatically converted into FHIR R4 resources, making it immediately interoperable.

A primary clinical use case for this workflow is the initial patient presentation (Erstvorstellung), where clinicians often encounter complex, fragmented external medical histories. In this workflow, documents such as discharge letters and pathology reports are scanned or uploaded upon the patient's arrival. The framework runs in the background, extracting and structuring data in real-time while the physician conducts the anamnesis. This automation has the potential to reduce administrative burden, allowing the physician to maintain eye contact and focus on the patient interaction rather than manual data entry. By the time the physical examination is complete, the structured data is available, providing an immediate overview of the patient's history and facilitating the efficient creation of admission notes directly within the Hospital Information System (HIS).

**
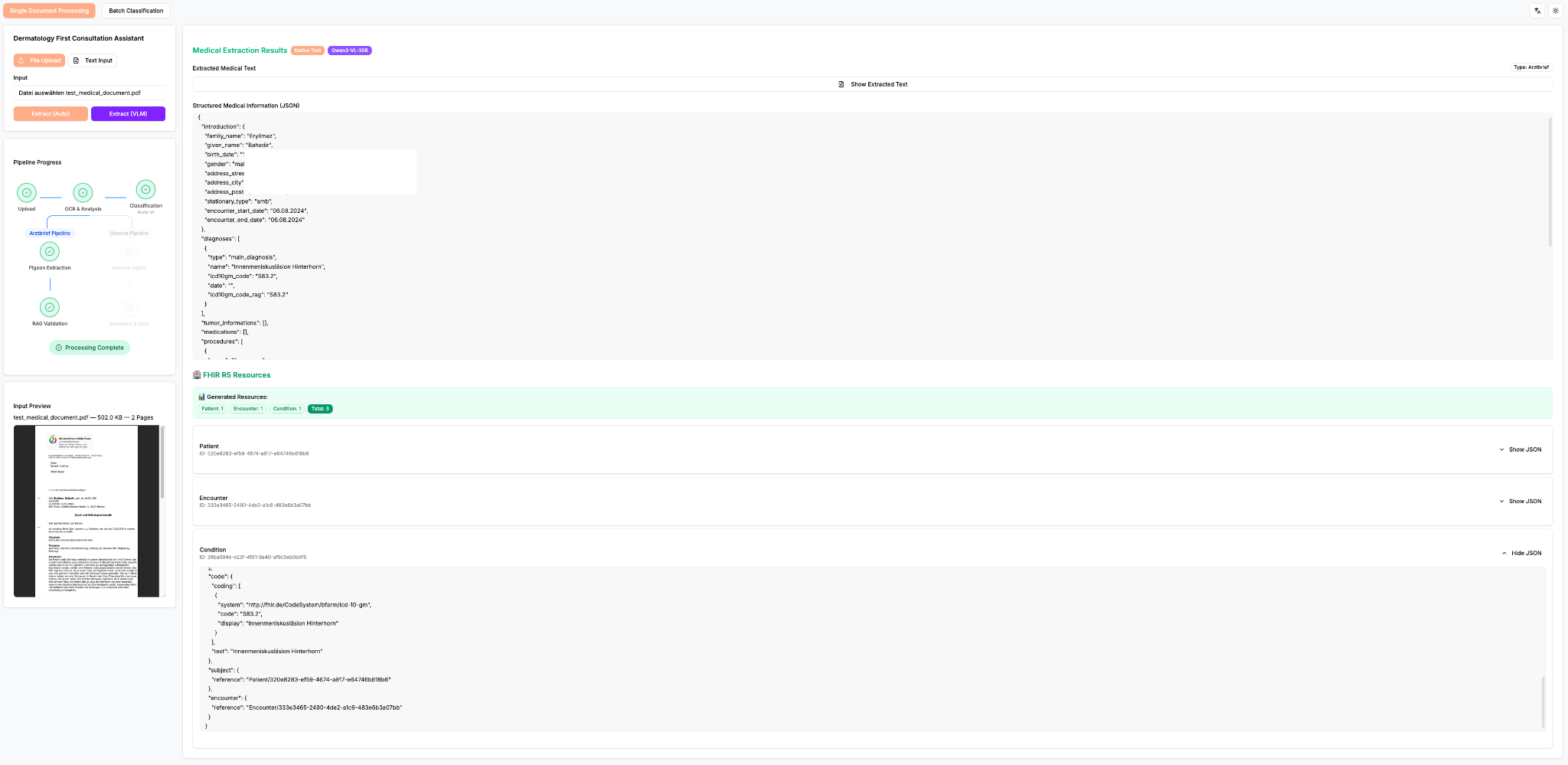
Figure B1: Interface of the prototype application.** The screenshot illustrates the end-to-end workflow for processing unstructured external medical documents during a patient encounter. The left panel manages file ingestion and tracks the status of the multi-stage pipeline (OCR/VLM text extraction, structural extraction via the fine-tuned PIGEON model (if classified as discharge letters), and RAG-based code correction). The main view displays the recovered raw text alongside the structured JSON output containing extracted clinical entities (e.g., demographics, diagnoses). The bottom section visualizes the automatic conversion of this structured data into HL7 FHIR R4 resources, facilitating direct integration with hospital information systems.

## C Training and Inference Parameters

### C.1 Training Methodology

The model was fine-tuned using the Google Gemma 3 27B instruction-tuned variant google/medgemma-27b-text-it as the base model. To ensure computational efficiency while maintaining performance, we utilized the Unsloth library with 4-bit quantization (QLoRA).

The training process employed Low-Rank Adaptation (LoRA) to update the model weights. We targeted all linear layers, including language, attention, and MLP modules, without applying dropout to the adapters. The model was trained with a context window of 8,192 tokens to accommodate long clinical texts.

Optimization was performed using the 8-bit AdamW optimizer with a constant learning rate schedule. To stabilize training on the 27B parameter model, we utilized a gradient accumulation strategy to achieve an effective batch size of 64. The training was configured to mask the user instructions, calculating loss solely on the model's responses (train_on_responses_only). All the parameters and values are demonstrated in Table C1.

| **Parameter** | **Value** | **Description** |
| --- | --- | --- |
| **Base Model** | medgemma-27b-text-it | Base checkpoint |
| **Quantization** | 4-bit (NF4) | Loaded via Unsloth |
| **Context Length** | 8,192 | Maximum sequence length |
| **LoRA Rank (r)** | 16 | Adaptation rank |
| **LoRA Alpha (alpha)** | 16 | Scaling factor (1:1 ratio with r) |
| **LoRA Targets** | All linear layers | Attention, MLP, and Language layers |
| **LoRA Dropout** | 0.0 | No dropout applied to adapters |
| **Learning Rate** | 2 x 10-5 | Constant schedule |
| **Optimizer** | AdamW (8-bit) | Memory-efficient optimizer |
| **Batch Size** | 4 | Per-device training batch size |
| **Grad. Accumulation** | 16 steps | Steps before backward pass |
| **Effective Batch Size** | 64 | 4 times 16 |
| **Max Grad Norm** | 0.3 | Gradient clipping threshold |
| **Weight Decay** | 0.01 | Regularization |
| **Warmup Steps** | 5 | Linear warmup |
| **Epochs** | 2 | Total training passes |
| **Seed** | 3407 | Random seed for reproducibility |

**Table C.1: Model configuration and fine-tuning hyperparameters.** The table details the architectural and training settings for the MedGemma-27b model, including 4-bit NF4 quantization, LoRA adapter configurations (rank 16, alpha 16), and optimization parameters (AdamW, learning rate 2 x 10-5) used to ensure efficient convergence and reproducibility.

### C.2 Inference Configuration

Inference was conducted using the vLLM high-throughput serving engine to ensure low-latency generation. The deployment utilized an asynchronous architecture allowing for concurrent request handling. We employed a deterministic decoding strategy with a low temperature (0.3) and a repetition penalty to prevent looping in clinical summaries, while top_p sampling was kept standard to maintain output coherence. The parameters are shown in Table D2.

| **Parameter** | **Value** | **Description** |
| --- | --- | --- |
| **Serving Engine** | vLLM | Version compatible with Gemma 2 |
| **Temperature** | 0.3 | Controls randomness (lower is more deterministic) |
| **Top-p (Nucleus)** | 0.95 | Cumulative probability cutoff |
| **Repetition Penalty** | 1.2 | Penalizes repeated tokens |
| **Max Output Tokens** | 8,192 | Maximum generation length |
| **Concurrency** | 10 | Max concurrent API requests |

**Table D.2: Inference configuration.** The table outlines the deployment settings used with the vLLM engine, including a temperature of 0.3 and a repetition penalty of 1.2, selected to ensure deterministic and coherent clinical summaries while maximizing throughput via asynchronous handling.

## D Medication and Lab Values Templates

The discharge letters analyzed in this study are archived as plain text. Although originally generated as PDF documents, they are retrieved in text format for processing. This conversion process introduces formatting discrepancies, particularly within structured sections such as medication lists and laboratory values. The extent of these layout alterations varies across different document templates.

### Medication Template 1: Compact List Representation

| Medikation:  Ramipril 5mg 1-0-0  Metoprolol 47.5mg 0-0-1 |
| --- |

### Medication Template 2: Enumerated Vertical Record

| Nr.  Medikament  Wirkstoff  Häufigkeit  Dosierschema  DF  Kommentar   1.  Ramipril 5mg  -    1-0-0  -  - |
| --- |

### Medication Template 3: Discharge Summary Context

| Medikation bei Entlassung:  Ramipril 5mg 1-0-0  Metoprolol 47.5mg 0-0-1   Die häusliche Medikation kann unverändert eingenommen werden: |
| --- |

### Medication Template 4: Therapeutic Recommendation Matrix

| Therapieempfehlung:  Wirkstoff  **St**ärke  Form  Morgens  Mittags  Abends  Einheit  Hinweise  Ramipril 5mg   1 0 0 |
| --- |

### Medication Template 5: Extended Long-term Medication

| Dauermedikation   Handelsname  Wirkstoff  Stärke  Form  Morgens  Mittags  Abends  Zur Nacht  Hinweise  Verordnungsgrund  Metoprolol 47.5mg    0 0 1 0 |
| --- |

### Medication Template 6: Simplified Medication Plan

| **Medikationsplan:   Ramipril 5mg 1-0-0   Metoprolol 47.5mg 0-0-1** |
| --- |

### Lab Values Template 1

| Laborparameter: Bestimmung Wert  Flag Status Vorwert vom Einheit Referenz  Hb 14.2  - gemessen 13.8 g/dl 13.5-17.5 |
| --- |

### Lab Values Template 2

| Laborparameter **bei** Aufnahme: **Bestimmung** Wert Einheit Flag Referenz vom Status  Hb 14.2 g/dl - 13.5-17.5 F |
| --- |

### Lab Values Template 3

| Labor: Bestimmung Wert Einheit Flag Referenz vom Status  Hb 14.2 g/dl - 13.5-17.5 F |
| --- |

### Lab Values Template 4

| Laborwerte **bei** Entlassung: **Bestimmung** Wert Einheit Flag Referenz vom Status  Hb 14.2 g/dl - 13.5-17.5 F |
| --- |

### Lab Values Template 5

| Laborparameter **bei** Entlassung: **Bestimmung** Wert Einheit Flag Referenz vom Status  Hb 14.2 g/dl - 13.5-17.5 F |
| --- |

## E Representative Error Examples from One-Shot Baseline Model

The following examples illustrate characteristic failure modes observed in the one-shot baseline model (GPT-OSS 120B) when extracting structured clinical information from German discharge letters. Across 884 evaluated samples, 97.2% of predictions contained no OPS procedure codes despite correct procedure name identification, and 63.1% had partially or completely empty ICD-10-GM codes. Three representative cases are shown below with the model prediction and ground truth in JSON format.

**Example 1 (Sample 25): Complete coding failure, all ICD and OPS codes empty**

**Prediction:**

{

"diagnoses": [

{"type": "main_diagnosis", "name": "Multifokales Lungensarkom", "icd10gm_code": ""},

{"type": "side_diagnosis", "name": "Muskelblutung aufgrund von Trauma", "icd10gm_code": ""},

{"type": "side_diagnosis", "name": "COPD mit akuter Verschlimmerung", "icd10gm_code": ""},

{"type": "side_diagnosis", "name": "Bronchialkarzinom mit kleinen Zellen", "icd10gm_code": ""},

{"type": "side_diagnosis", "name": "Transversale Fraktur des 8. Brustwirbels", "icd10gm_code": ""}

],

"free_text": {

"procedures": [

{"procedure_name": "CT Thorax mit Kontrastmittel", "procedure_code": "", "code_type": ""},

{"procedure_name": "CT Abdomen mit Kontrastmittel", "procedure_code": "", "code_type": ""},

{"procedure_name": "Diagnostische Koloskopie bis Zaekum", "procedure_code": "", "code_type": ""},

{"procedure_name": "Standard-OP AC", "procedure_code": "", "code_type": ""}

]

}

}

**Ground Truth:**

{

"diagnoses": [

{"type": "main_diagnosis", "name": "Lungensarkom mehrere Teile ueberlappend", "icd10gm_code": "C34.8"},

{"type": "main_diagnosis", "name": "Muskelhaematom onA", "icd10gm_code": "T14.05"},

{"type": "main_diagnosis", "name": "COPD mit akuter Exazerbation, FEV1 >=50% <70%", "icd10gm_code": "J44.12"},

{"type": "main_diagnosis", "name": "kleinzelliges Bronchialkarzinom", "icd10gm_code": "C34.9"},

{"type": "side_diagnosis", "name": "Proc. transversus Fraktur BWK 8", "icd10gm_code": "S22.04"}

],

"free_text": {

"procedures": [

{"procedure_name": "CT Thorax mit Kontrastmittel", "procedure_code": "3-222", "code_type": "ops"},

{"procedure_name": "Computergestuetzte Bilddatenanalyse 3D", "procedure_code": "3-990", "code_type": "ops"},

{"procedure_name": "CT Abdomen mit Kontrastmittel", "procedure_code": "3-225", "code_type": "ops"},

{"procedure_name": "Standard OP AC", "procedure_code": "1500-001", "code_type": "ops"},

{"procedure_name": "Diagnostische Koloskopie: Total, bis Zaekum", "procedure_code": "1-650.1", "code_type": "ops"}

]

}

}

**Example 2 (Sample 654): Partial ICD failure with incorrect sub-digits (C49.1 vs C49.2)**

**Prediction:**

{

"diagnoses": [

{"type": "main_diagnosis", "name": "Chronisch-infektioese Wundheilungsstoerung", "icd10gm_code": ""},

{"type": "side_diagnosis", "name": "Weichteiltumor, myxoides Liposarkom", "icd10gm_code": "C49.1"},

{"type": "side_diagnosis", "name": "Staphylococcus-caprae-Infektion", "icd10gm_code": ""},

{"type": "side_diagnosis", "name": "Iatrogene Immunkompromittierung (Strahlen/Zyto/MTX)", "icd10gm_code": ""}

]

}

**Ground Truth:**

{

"diagnoses": [

{"type": "main_diagnosis", "name": "Re-Re-WHS infektioes", "icd10gm_code": "T81.4+"},

{"type": "main_diagnosis", "name": "Myxoides Liposarkom dist. Oberschenkel re.", "icd10gm_code": "C49.2"},

{"type": "side_diagnosis", "name": "Staph. caprae als Erreger", "icd10gm_code": "B95.7!"},

{"type": "side_diagnosis", "name": "Immunkompromittierung nach Bestrahlung/Chemo/MTX", "icd10gm_code": "D90"}

]

}

**Example 3 (Sample 114): Complete OPS procedure code failure with ICD sub-digit errors**

**Prediction:**

{

"diagnoses": [

{"type": "main_diagnosis", "name": "Linksseitiges Mammakarzinom", "icd10gm_code": "C50.9"},

{"type": "side_diagnosis", "name": "Oesophagitis durch Reflux", "icd10gm_code": "K21.0"},

{"type": "side_diagnosis", "name": "Rezidivierendes Fieber", "icd10gm_code": "R50.9"},

{"type": "side_diagnosis", "name": "Obesity class I", "icd10gm_code": "E66.0"}

],

"free_text": {

"procedures": [

{"procedure_name": "BET/Ablatio mit SLNE, SS, Axilla-Dissektion", "procedure_code": "", "code_type": "ops"},

{"procedure_name": "Primaere rekonstruktive Mammoplastik mit Alloprothese", "procedure_code": "", "code_type": "ops"}

]

}

}

**Ground Truth:**

{

"diagnoses": [

{"type": "main_diagnosis", "name": "Boesartige Neubildung: Brustdruese links", "icd10gm_code": "C50.9"},

{"type": "main_diagnosis", "name": "Schuerfwunde Knie", "icd10gm_code": "S80.81"},

{"type": "side_diagnosis", "name": "Reflux im Oesophagus", "icd10gm_code": "K21.9"},

{"type": "side_diagnosis", "name": "Sonstiges Fieber (Rez.)", "icd10gm_code": "R50.88"},

{"type": "side_diagnosis", "name": "Adipositas (BMI 31.89)", "icd10gm_code": "E66.00"}

],

"free_text": {

"procedures": [

{"procedure_name": "BET oder Ablatio mit SLNE, SS, Axilla", "procedure_code": "2400-600", "code_type": "ops"},

{"procedure_name": "Sondenmessung SLNE", "procedure_code": "3-760", "code_type": "ops"},

{"procedure_name": "Mammographie: Praeparatradiographie", "procedure_code": "3-100.1", "code_type": "ops"},

{"procedure_name": "Plastische Rekonstruktion Mamma mit Alloprothese", "procedure_code": "5-886.31", "code_type": "ops"}

]

}

}

##

##

## F OPS Error Analysis

We analyzed the human-validated PIGEON predictions across this hierarchy to characterize where the model diverges from annotator-corrected codes. Strict full-code agreement was 61.3%, OPS chapter-level agreement was 70.1%, and the procedure description in the discharge text was correctly extracted in 76.0% of cases. Inspection of the wrong predictions identified OPS-catalogue ambiguity as the dominant pattern. For several clinically common procedures, the model assigned an OPS code from a different chapter than the annotator. The predicted codes were not random but semantically related to the documented procedure, indicating that the model recognized the broad procedure type but defaulted to an adjacent OPS chapter. Our error analysis also identified a distributional shift in OPS chapters between the randomly sampled training corpus and the oncology-focused evaluation discharge letters, which is consistent with this tendency to favor semantically related chapter choices over the annotator's preferred encoding.

## G Operational Definitions of FHIR resources

Each patient journey in the study cohort included the FHIR resources specified in [Table](#ts5oskuqj7oo)2:

| Resource Type | Description |
| --- | --- |
| **Patient** | Demographic and administrative information about the individual. |
| **Encounter** | An interaction between a patient and healthcare provider(s), typically corresponding to a hospital visit or consultation. |
| **Condition** | A clinical condition, diagnosis, problem, or other event that the patient has or is at risk of developing. This includes cancer diagnoses. |
| **Procedure** | An action that is or was performed on or for a patient (e.g., surgery, radiotherapy). |
| **Observation** | Measurements, simple assertions, or estimates made about a patient (e.g., lab values, vital signs, tumor informations, body weight, height). |
| **Medication** | Specific medication/vaccine type |
| **MedicationStatement** | A record of a patient's medication usage, including prescription and non-prescription drugs. |

**Table G.1: Operational definitions of FHIR resources constituting the study cohort's clinical data corpus.** The table outlines the specific resource types extracted from the in-house server, encompassing demographic, administrative, and clinical interaction data used to model the patient trajectory.
